# Hybrid Markov-mass action law model for cell activation by rare binding events: Application to calcium induced vesicular release at neuronal synapses

**DOI:** 10.1038/srep35506

**Published:** 2016-10-18

**Authors:** Claire Guerrier, David Holcman

**Affiliations:** 1 Computational Biology and Applied Mathematics, Ecole Normale Supérieure Paris, France; 2 DAMTP and Churchill College, University of Cambridge, Cambridge CB30DS United Kingdom

## Abstract

Binding of molecules, ions or proteins to small target sites is a generic step of cell activation. This process relies on rare stochastic events where a particle located in a large bulk has to find small and often hidden targets. We present here a hybrid discrete-continuum model that takes into account a stochastic regime governed by rare events and a continuous regime in the bulk. The rare discrete binding events are modeled by a Markov chain for the encounter of small targets by few Brownian particles, for which the arrival time is Poissonian. The large ensemble of particles is described by mass action laws. We use this novel model to predict the time distribution of vesicular release at neuronal synapses. Vesicular release is triggered by the binding of few calcium ions that can originate either from the synaptic bulk or from the entry through calcium channels. We report here that the distribution of release time is bimodal although it is triggered by a single fast action potential. While the first peak follows a stimulation, the second corresponds to the random arrival over much longer time of ions located in the synaptic terminal to small binding vesicular targets. To conclude, the present multiscale stochastic modeling approach allows studying cellular events based on integrating discrete molecular events over several time scales.

Cellular activation is described by the binding of few diffusing molecules to specific small and hidden targets. What is the time scale of a cell response, triggered by rare molecular events? Such a process requires exploring microdomains at a nanometer precision. Examples are vesicular fusion triggered by the binding of calcium ions, detection of a morphogen concentration by a growth cone, molecular events underlying the induction of synaptic plasticity at neuronal synapses or activating cellular check points where molecular events are transformed into a cellular decision.

Traditional chemical kinetics^1^ are based on mass-action laws or reaction-diffusion equations, but this is a deficient description of stochastic chemical reactions in micro-domains, where only a small number of molecules are involved^2^,^3^,^4^. Several models were developed to describe stochastic reactions, such as Markov reaction-diffusion equations based on the joint probability distribution for the concentration of bound and unbound reactants, leading to coupled systems of reaction-diffusion^5^. Another direction involves the Narrow Escape Theory^5^ to coarse-grain the binding and unbinding reactions to the time scale of diffusing reactant in and out of a binding site. This approach reduces the classical reaction-diffusion approach to a Markovian jump process description of the stochastic dynamics for the binding and unbinding of molecules. The Markovian approximation^1^,^6^ can be used for example to obtain predictions for the rate of molecular dynamics underlying spindle assembly checkpoint during cell division^7^ or the probability that a messenger RNA escapes degradation through binding a certain number of microRNAs. The Markovian approach can also be used to compute the mean time the number of bound molecules reaches a threshold, called the mean time to threshold theory (MTT), for characterizing the stability of activation^6^.

However, none of the approaches described above have been used to model the interaction between a large bath of particles modeled by a continuous dynamic and a stochastic dynamic induced by rare events where few particles bind to small targets to trigger a response.

The goal of this report is to introduce a novel hybrid model and to provide an application to synaptic physiology for computing the probability of vesicular release following calcium influx. Calcium ions induce vesicular release when few of them accumulate underneath a vesicle. This accumulation can follow the direct influx at a short time scale or can be due to ions originating from the pre-synaptic terminal with a longer time scale. In all cases, triggering a vesicle release is a rare molecular event described as the accumulation of few ions at a single protein location^8^. However, due to instrumental limitations, it is not yet possible to measure calcium dynamics at a nanometer level at neuronal synapses. Yet, synchronous and asynchronous synaptic release might depend on calcium dynamics and we provide here several predictions using the present hybrid stochastic model of chemical reactions, that we compare to Gillespie simulations. The proposed mechanism of asynchronous vesicular release that we found here clarifies the role of calcium ions during short-term synaptic plasticity^9^.

## Results

### Hybrid Markov-mass action model

**Figure 1:**
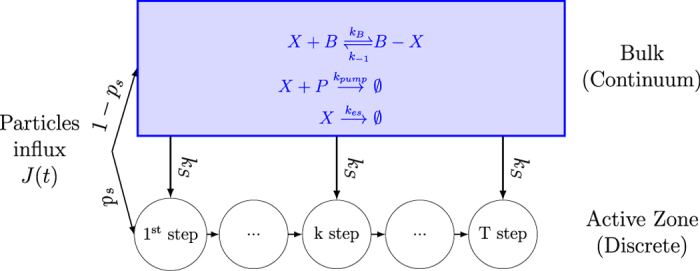
Schematic representation of the coupled Coarse-grained Markov to mass action. Particles enter at a source point with a flux *J*(*t*) and are then divided between the target sites, with probability *p*_*s*_ (Eq. 2) and the bulk with probability 1 − *p*_*s*_. In the bulk, particles *R* react with the substrate *S*, modeled by the classical mass action equations, with forward rate *k*_0_, and backward rate *k*_−1_. The escape rate from the bulk is *k*_*es*_. Particles located in the bulk can also bind to the targets with constant rate *k*_*T*_. The overall scheme represents the coupled mass action equations (in the bulk) with a Markov chain and the ensemble models the arrival of *T* particles to a single target.

Reactant molecules *R* are modeled as Brownian particles, entering a domain Ω through localized point sources with an inward flux *J*(*t*) at each single source. The entering Brownian particles can reach any target site to activate them. After particles enter, they can either hit a small target or reach the bulk, where they are lost in the undifferentiated state consisting in many particles. In the bulk, the reactant particles *R* can either interact with a substrate S, leave the bulk with a rate *k*_*es*_ or bind a target site with rate *k*_*T*_, as shown in Fig. 1. The arrival of *T* particles at a single target is the event that we are interested in, as it models a cellular activation from a molecular event. As we shall see here, the Brownian reactant particles and the small targets can be described by a coarse-grained model, where the mass action description (for the continuum limit) is coupled to a finite state Markov chain (Fig. 1).

**Figure 2:**
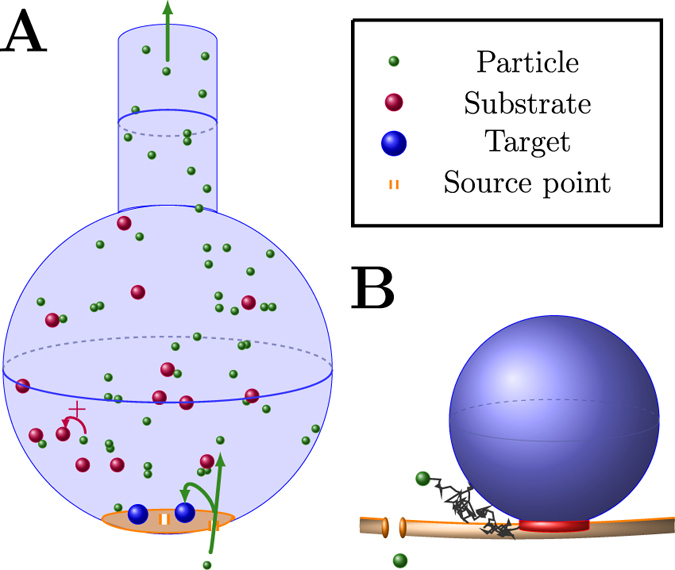
Representation of the pre-synaptic terminal. (**A**) A Brownian particle (green) entering the pre-synaptic terminal either reaches a target (blue) located near the source points (orange) or reaches the bulk. In the bulk, the particle can bind to a substrate or leave the domain. (**B**) Schematic description of the arrival of a particle to a target site. The Brownian particles enter through source points. They can reach the small ribbon (red) located underneath a target (blue). The arrival of *T* particles to the ribbon is needed to activate the target.

For the application to vesicular release described in the second part, the bulk represents the pre-synaptic terminal (Fig. 2A), and *N*_*T*_ the number of small target sites (red ribbon in Fig. 2B) hidden underneath a ball representing a vesicle.

The flux *J*(*t*) of particles enters the domain at a source point *x*. The entering particles can reach a small target with a splitting probability *p*_*s*_(*x*) or go to the bulk with probability 1 − *p*_*s*_. A particle entered at point *x* that reaches one of the *N*_*T*_ target sites, will reach specifically site *i* with probability *q*(*x*, *i*), for *i* = 1..*N*_*T*_ (see the Methods section for the description). Thus the flux of ions arriving at a target site *i* is the sum of the conditional fluxes over the ensemble of point source locations 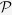:


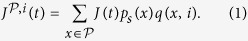


We recall that the splitting probability *p*_*s*_(*x*) for a Brownian particle to reach the small ribbon below a ball (Fig. 2B) before entering the bulk, when balls are distributed on a square lattice, has been estimated previously using conformal mapping methods and asymptotic approximations^10^:


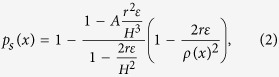


where *r* is the size of the ball, *H* is half the distance between two targets, *A* = 9.8, *ρ*(*x*) is the distance between the point source and the closest target site, and *ε* is the height of the ribbon representing the target site. This probability accounts for the particular geometry of the target and depends on the relative distance between the targets and the source points^10^.

So far, we discussed the direct arrival of a particle from a point source. We now consider the arrival of a Brownian particle coming from the bulk to one of the small targets. This arrival to a small ribbon (red in Fig. 2B) is Poissonian with a rate (computed in ref. 10)


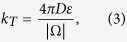


where *D* is the particle diffusion coefficient.

We shall now present the set of equations that describe the arrival of a few number of particles to the target either coming directly from the point source or from the bulk. For each target site *i* and for a given distribution *P* of source points, the probability *Pr*^*i*^{*k*, *t*} that *k* particles are bound at time *t* is computed from a Markov model: once a particle binds to a target, it cannot unbind, and thus the transition probability from the states *k* − 1 to *k* (bound particles), between *t* and *t* + Δ*t*, is due to the fluxes of particles from the point sources and from the bulk:


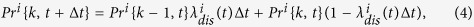


where 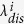 represents the arrival rate of particles to the target:


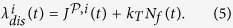


The first term 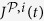 in the right-end side of the rate equation 5 represents the cumulative flux of particles reaching target site *i* summed over all point sources. It represents the amount of particles reaching target *i* directly after their entry through a source point. The second term *k*_*T*_*N*_*f*_ (*t*) represents the fraction of particles originating from the bulk that will bind to the target sites. Here *N*_*f*_ (*t*) is the number of free particles at time *t* in the bulk, and *k*_*T*_ is the arrival rate of particles located in the bulk. The rate *k*_*T*_ is computed using the Mean First Passage Time theory^10^, and when the target size is small compared to the size of the bulk, the binding events are rare and thus can be modeled as Poissonian rate which is the reciprocal of the mean first time for a particle to reach the target^5^,^10^. These two terms account for the targets geometry, as well as their relative positions with respect to the source points 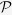.

We shall now described the model of activation. A target is considered to be activated when there are exactly *T* bound particles. We obtain for each target 1 ≤ *i* ≤ *N*_*T*_, the Markov chain for the probabilities 
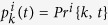
,


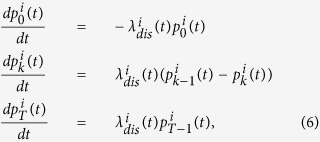


where the final equation for *k* = *T* describes the absorbing state for the probability 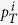. The initial condition at time *t* = 0 is


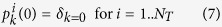


and the normalization condition is


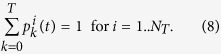


We can now introduce the time 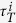 to a threshold *T* for target *i*, when the distribution of point source is 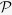. This time corresponds to the first time that there are exactly *T* particles at target *i*. It is also the last passage time of a particle when *T* − 1 particles are already at the binding sites. The distribution of time 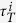 is called the activation time for the target *i* and is given by


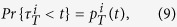


hence the probability density function 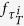 of the activation time is defined by


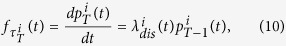


and the mean release time 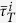 is thus


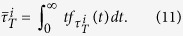


To conclude this section, we have presented here a Markov chain that counts exactly *T* binding events. We note that the particles originate from two sources: either from the bulk or a transient influx. We shall introduce in the next section a new set of equations to couple the Markov chain and the density of free particles *N*_*f*_ (*t*) that can further interact with a varying population of substrates *S*.

### The mass action law equations for reactant particles interacting with a substrate in the bulk

The reactant particles in the bulk can interact with a substrate according to the chemical reaction


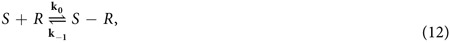


where *k*_−1_ (resp. *k*_0_) is the backward (resp. forward) rate. We determine the mass-action equations for the number of free *N*_*f*_ (*t*), and bound *N*_*b*_ (*t*) particles. The total number of substrate sites *S*_*tot*_ is fixed, and at time *t*, the number of available sites is *S*_*tot*_ − *N*_*b*_(*t*). The particles can escape the domain Ω at a Poissonnian rate *k*_*es*_, and bind to a target site with a rate *k*_*T*_. We note that the probability that target *i* is free, i.e. not activated, is given by


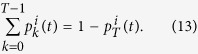


In summary, *N*_*f*_ and *N*_*b*_ satisfy the mass action equations:


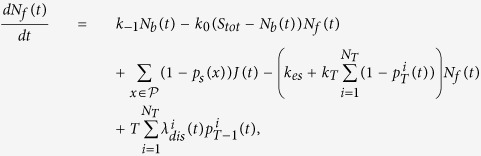



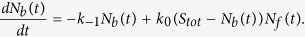


The first two terms in the first equation represent the classical unbinding and binding of molecules to buffers. The third term represents the fraction of particles directly entering the bulk from the initial influx. The fourth term corresponds to particles that leave the bulk or bind to a free target (we assume that the escape rate *k*_*es*_ is fast compared to the binding rates). Finally the last term accounts for the release into the bulk of the *T* particles bound to a target, following the target activation. Indeed, after activation, the *T* bound particles are released into the bulk, leading to an increase of the free particles *N*_*f*_ by a jump event of *T* particles (Eq. 10). The second equation is the classical mass action law for the number of bound buffers.

Interestingly, the system of equations 6, 7, 8, 9, 10, 11, 12, 13, 14 can be decoupled and resolved. We use a direct integration between *t*_0_ and *t* for the probability 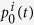 and then inductively derive all probabilities 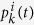. Starting with equations 6, we get


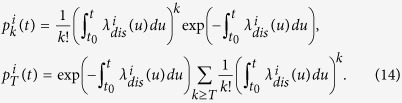


The solutions can then be directly injected into the mass action system 14. To conclude, equations 6, 7, 8, 9, 10, 11, 12, 13, 14 involve the coupling of the probability 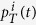 to the mass action equations and the ensemble constitutes a coupled set of Markov-mass action equations, that describes the cumulative binding of *T* particles, interpreted as activation.

We shall now provide an application to neurobiology about calcium signaling at single neuronal synapses. There are indeed 10^11^ neurons in a human brain, and each has a mean of 10^3^ synapses. Synapses are thought to code part of the memory, mainly by modulating the neuronal response through time variations in the release of vesicles. We will study now some aspects of the stochastic release of a vesicle from the accumulation of *T* = 5 calcium ions to key proteins. This example illustrates the theory presented above in the context of synaptic activation from rare molecular binding events.

### Dynamics of vesicular release at neuronal synapse

The pre-synaptic terminal of a neuronal cell contains a large amount of vesicles that can be randomly released following an action potential, characterized by a release probability^11^. However, the release mechanism is not well understood and computing the release probability remains a challenge^12^,^13^,^14^,^15^. The mechanism is described as follows: after an action potential invades the pre-synaptic terminal, voltage-gated calcium channels (VGCCs) open, leading to a calcium influx into the pre-synaptic domain^8^,^16^,^17^. When several diffusing calcium ions have succeeded to find small molecular targets, which form a complex of molecular machinery (such as synaptotagmin and any other key molecules) located underneath a vesicle, these molecules are changing their conformation, activating the fusion of the vesicle with the cell membrane and thus triggering the release of neurotransmitters^11^. This is a key step of neuronal communication.

At the same time, calcium ions can bind and unbind to buffer molecules located in the bulk of the pre-synaptic terminal, or exit at the end of the terminal. The success of vesicular release depends on rare events: the arrival of diffusing calcium ions to the small target molecules^18^,^19^,^20^. Ions are either coming directly from the initial entering calcium flux or from ions located the bulk. Vesicular release also depend on the relative position between vesicles and the VGCCs and on their organization on the surface^10^.

To investigate the time distribution of vesicular release triggered by an accumulation of *T* = 5 calcium ions at a ribbon (Fig. 2B), we solved numerically the system of ODE Eqs 6, 7, 8, 9, 10, 11, 12, 13, 14 using Matlab (see the Methods section for the description).

**Figure 3:**
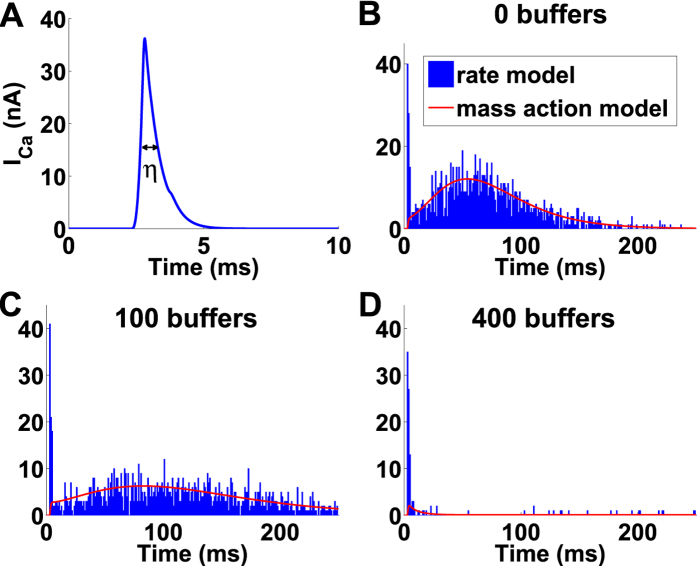
Calcium time course in the pre-synaptic terminal and vesicular release activation. Channels are uniformly distributed around the vesicles. (**A**) Calcium current entry, simulated by a classical Hodgkin-Huxley model^21^. (**B**–**D**) Histogram of vesicular release time for the Markov-mass action model (red) and the Poissonian rates (blue) when there are zero (**B**), 100 (**C**), and 400 buffer sites (**D**). The continuous red curves are solutions of the hybrid model 6–14 and are compared to Gillespie simulations realized using Poissonian rates.

The initial influx of calcium ions through a VGCC *J*(*t*) is simulated using a simplified Hodgkin-Huxley model for calcium current^21^: the membrane voltage depolarizes following an action potential, modeled by a transient current *I*_*app*_(*t*). The induced calcium current lasts 2.75 ms, with a maximal value of *I*_*Ca*,max_ = 36.2 nA (Fig. 3A). The total charge *Q* = 0.025 fC corresponds to an entry of 80 calcium ions (Methods section). We consider that vesicles are located on a square lattice and that channels are uniformly distributed. The probabilities *q*(*x*, *i*) are estimated numerically using Brownian simulations (Methods section). All parameters used for the simulations are described in Table 1.

To confirm the result of this novel Markov-mass action model (red curves in Fig. 3B), we propose an alternative approach using a Gillespie algorithm based on Poissonian rates. The Gillespie’s simulations track rare binding events to small targets, which is well approximated by single exponential distribution the rate of which is the mean first binding time^22^. Due to the small size of the binding sites compared to the volume of the domain (Table 1), the motion of calcium ions in the bulk -the binding on the SNARE Complex, the binding on buffers an the escape from the domain- can all be approximated by a rate process, computed from the Narrow Escape Theory^10^,^23^. This approximations allows us to use the Gillespie algorithm (blue traces in Fig. 3B), using the rates presented in Table 1 (see the Methods section for a description). We found that the two models quite different in nature agree together (Fig. 3B).

When there are no buffers in the bulk (Fig. 3B), the time distribution of vesicular release is bimodal with two consecutive stages: a short time period following the immediate entrance of calcium ions. In that case, vesicular release is triggered by ions that are directly coming from the channel influx. The second regime characterized by a broader peak is induced by the random arrival of calcium ions from the bulk to the target sites, until *T* sites per vesicle are occupied. The large release time distribution is due to the long time scale of arrival for ions that could be bound and need to diffuse to the small targets. Using formula 3, we obtain for a volume of 1 *μm*^3^, *D* = 20 *μm*^2^/*s* and *ε* = 0.001 *μm*, a mean time 
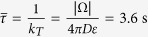
. This time scale is much longer than the initial calcium transient from the channels and explains the long residual effect of calcium on vesicular release.

To further clarify the origin of the residual (second) peak, we tested numerically the effect of considering buffers in the synaptic bulk at various concentrations. Their role is to buffer the calcium ions in the bulk. We found that increasing the number of buffers abolishes the second peak (Fig. 3B–D), confirming that it is due to the arrival of free calcium ions located in the bulk.

We conclude this report with the following prediction: the diffusion time course in the bulk (which is modeled as a ball of radius 600 nm), where target binding sites are located underneath vesicles of size 20 nm leads to a residual vesicular release that peaks around 60 ms. However, in the case of a high buffer concentration, because the mean unbinding time to buffer is 1/*k*_−1_ = 2 *s*, at the time scale of tens of ms most ions are still buffered and the second peak disappears (Fig. 3C,D) (the escape rate is 1/*k*_*es*_ = 0.7 s). However, we can still observe rare events in the distribution tail due to the arrival of free ions from the bulk.

## Discussion and Conclusion

We presented here a hybrid model that connect a Markov process to a mass action model called the Markov-mass action model. The aim is to study the coupling between a continuum ensemble of particles that participate into a small number of rare events, representing the binding of a small and finite tiny subset of these Brownian molecules to small targets. It was already noticed that discrete-state stochastic models for particles are not well approximated by continuum equations^24^,^25^. The present model is also quite different from previous numerical simulation efforts to generate a finite number of Brownian trajectories from a continuum ensemble^26^,^27^.

To illustrate the applicability of this method, we model vesicular release at synapses from calcium dynamics, where we found a first peak at short time scale (less than 10 ms) induced by the direct entrance of calcium ions, followed by a second peak, which is due to the return of calcium from the bulk to the small hidden targets, a process that also depends on the interaction with buffer molecules and can last hundreds of milliseconds. The emergence of these two phases can possibly explain asynchronous quantal release, without introducing any additional time constant at a molecular level. The present approach could also be used to describe short-term plasticity at a molecular level^20^,^28^ and to model the noise inherent to synaptic dynamics^29^.

## Methods

### Influx of particles

The initial influx of particles through a channel following an action potential is computed using a simplified Hodgkin-Huxley model. The membrane depolarization following an action potential is modeled using an applied current *I*_*app*_ = 50 mV during 1 ms. The equations are summarized below


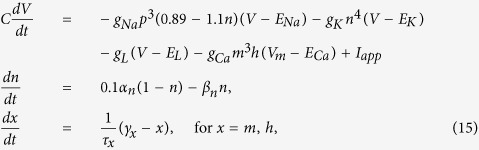


where


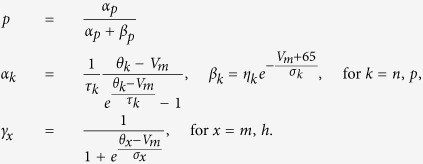


The initial values *V*_0_, *n*_0_, *m*_0_, *h*_0_ are the equilibrium solutions of the ODE. They are summarized with all the parameters in Table 2. The induced calcium current lasts 2.75 ms, with a peak value *I*_*Ca*,max_ = 36.2 nA (Fig. 3A).

### Numerical estimation of the splitting probability between targets

**Figure 4:**
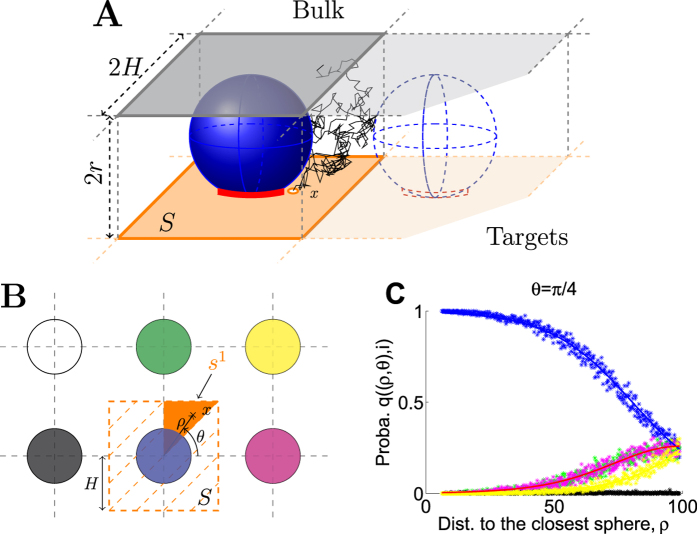
Numerical estimation of the arrival probability to a target. When Vesicles are distributed on a square lattice, and channels are positioned on squares *S* of length 2*H* around them (**A,B**). Brownian particles (calcium ions) enter through channels located at point *x* on the square *S* (orange in **A**). They enter the bulk when they reach the upper face (gray square in **A**) of the rectangular cuboid around a sphere with lower face *S* and height 2*r*. Each square *S* can be subdivided into triangular subunits *s*^*i*^ (**B**), where Brownian simulations are used to estimate the probability that a particle reaches the closest target (blue in **B,C**), the second closest (green or magenta), and so on. The length is *H* = 70 nm (Table 1) and we show in (**C**) the results for *θ* = *π*/4. The color code in (**B**,**C)** is identical.

In this section, we describe the numerical simulations that we used to estimate the probability to distributed an ions among one of the *N*_*T*_ target sites (vesicles), distributed on a square lattice. There exactly *N*_*c*_ source points (channels) distributed in a square *S* of length 2*H* near the targets^10^ (Fig. 4A,B and Table 1).

After particles either enter the bulk or bind to one of the targets. In the latter case, using Brownian simulations we determined the target. For that, we subdivide each square *S* into 8 sub-triangles (*s*^*i*^)_*i*=1...8_ (Fig. 4B). Using symmetry we restrict our analysis to triangle *s*^1^. For a point source *x *∈ *s*^1^ (Fig. 4B), we estimate the probability *q*(*x*, *i*_1_) that the entering particle binds to the closest target *i*_1_, (blue in Fig. 4B), to the second closest *i*_2_, *q*(*x*, *i*_2_) (green or magenta) and so on, with respect to the distance *dist*(*x*) to the closest vesicle. The normalization identity is 
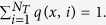


The exact numerical procedure is the following: To compute the distribution *q*(*x*, *i*), we further decompose the triangle *s*^1^ into blocks parameterized by polar coordinates (*ρ*_*k*_, *θ*_*k*_)_*k*_. We generated 200 runs for each block *k* and fitted the results using a routine procedure in Matlab (Fig. 4, the color code in B and C are the same). We neglect the probability to bind to the 5th-closest target and anyone further away. This numerical result allows to compute the probability *q*(*x*, *i*) to reach specifically the target site *i*, when starting from point *x*. Finally, the fraction of particles entering through a channel positioned at *x*, that reaches a target *i*, is *p*_*s*_(*x*)*q*(*x*, *i*), and the fraction of flux coming from a channel located at *x* and arriving to target *i* is *J*^*i*^(*x*, *t*) = *J*(*t*)*p*_*s*_(*x*)*q*(*x*, *i*).

### Mass action model

We solve numerically the coupled system of ODE Eqs 6, 7, 8, 9, 10, 11, 12, 13, 14 for different numbers of buffer sites and for a uniform distribution of channels using Matlab and Monte Carlo simulations. For a given number of buffer sites and a given positioning of the *N*_*c*_ channels, we solved the system using a Matlab solver. To get the results for a uniform distribution of channels (Fig. 3B–D), we perform Monte Carlo simulations (150 realizations).

### Stochastic simulations based on the Gillespie algorithm

The stochastic model is a Gillespie algorithm based on Poissonian rates. The arrival time of a Brownian particle to a small hole is well approximated by a Poisson process and the rate is the reciprocal of the mean first passage time. In our model, the arrival of a Brownian particle to a small hole can represent binding to buffers, escape from the domain and binding to a target site. We shall now present the rates used in simulations.

The buffers are positioned at independent sites, modeled by small spheres. The mean first arrival time 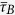 of a Brownian particle to a small spherical target of radius *r*_*B*_ in the domain Ω, with diffusion coefficients *D* for the particle and *D*_*B*_ for the buffer, is given by^23^


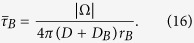


The release rate to a buffer site is Poissonian with backward rate *k*_−1_ (Table 1). The domain is described in Fig. 2 and consists of a head, of volume |Ω_*h*_| and a cylindrical neck with radius *r*_*neck*_ and height *l*_*neck*_ (Table 1). The escape at the end of the domain is Poissonian, with rate the reciprocal of the mean escape time^23^, given by


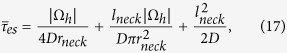


where *D* is the diffusion coefficient. Finally, the arrival of a particle to a small ribbon (colored in red in Fig. 2B) is Poissonian with rate given in Eq. 3. These arrival times are exponentially distributed with rates equal to the reciprocal of the binding time


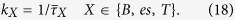


when there are *N*_*f*_ independent Brownian particles in the domain, the rate constant for the first binding event is


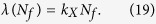


The conditional probability that a binding event *T*_*b*_ occurs between time *t* and *t* + Δ*t*, when there are *N*_*f*_ particles is


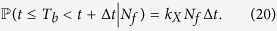


After entry through channels, particles either bind to the targets with probability *p*_*s*_ Eq. 2, or go to the bulk with probability 1 − *p*_*s*_. Using a Gillespie algorithm and the rates described above, we compute the time course of the number of particles in the bulk *N*_*f*_, and the time distribution of vesicular release (see also Table 1).

## Additional Information

**How to cite this article**: Guerrier, C. and Holcman, D. Hybrid Markov-mass action law model for cell activation by rare binding events: Application to calcium induced vesicular release at neuronal synapses. *Sci. Rep.* **6**, 35506; doi: 10.1038/srep35506 (2016).

## Figures and Tables

**Table 1:** Biophysical and geometrical parameters used in simulations.

Parameter	Description	Value
|Ω|	Volume of the pre-synaptic terminal	1 *μm*^3 ^30
*N*_*B*_	Buffer quantity	[0–700]
*r*_*B*_	Radius of the buffers binding site	0.001 *μm*^31^
*r*	Radius of the vesicle	0.02 *μm*^32^
*ε*	Height of the ribbon target	0.001 *μm*
*r*_*neck*_	Radius of the pre-synaptic neck	0.1 *μm*^32^
*l*_*neck*_	Length of the pre-synaptic neck	0.15 *μm*^32^
*N*_*T*_	Number of vesicles	8^32^
*N*_*c*_	Quantity of calcium channels	3
# Ca	# Ca entered after an AP through one channel	80^13^
*D*	Calcium diffusion coefficient	20 *μm*^2^*s*^−1 ^33
*D*_*B*_	Buffer diffusion coefficient	20 *μm*^2^*s*^−1 ^13
*k*_−1_	Buffer unbinding constant	500 ms
*τ*_*B*_	Buffer mean binding time	1.8 sec (computed from eq. 16)
*k*_0_ = (1)/(*τ*_*B*_)	Rate of binding to buffers	0.6 sec^−1^
*τ*_*T*_	Mean time to bind to a ribbon target	3.6 sec (computed from eq. 3)
*k*_*T*_ = (1)/(*τ*_*T*_)	Rate of binding to targets	0.3 sec^−1^
*τ*_*es*_	Mean time to leave through the neck	0.7 sec (computed from eq. 17)
*k*_*es*_ = (1)/(*τ*_*es*_)	Rate of leaving through the neck	1.5 sec^−1^

Extracted from literature for the Parallel fiber to Purkinje cell synapse, and estimated from the model.

**Table 2:** Hodgkin-Huxley parameters.

Parameter	Description	Value
*C*	Capacitance	1 *μ*F.cm^−2^
*g*_*Na*_	Conductance of *Na*^2+^-current	120 mS.cm^−2^
*E*_*Na*_	Equilibrium potential of *Na*^2+^-current	50 mV
*τ*_*p*_	Parameter for *p*	10 ms
*θ*_*p*_	Parameter for *p*	−40 mV
*η*_*p*_	Parameter for *p*	4
*σ*_*p*_	Parameter for *p*	18
*g*_*K*_	Conductance of *K*^+^ -current	36 mS.cm^−2^
*E*_*K*_	Equilibrium potential of *K*^+^ -current	−77 mV
*g*_*L*_	Conductance of leak current	0.3 mS.cm^−2^
*E*_*L*_	Equilibrium potential of leak current	−54.4 mV
*τ*_*n*_	Parameter for *n*	10 ms
*θ*_*n*_	Parameter for *n*	−55 mV
*η*_*n*_	Parameter for *n*	0.125
*σ*_*n*_	Parameter for *n*	80
*g*_*Ca*_	Conductance of *Ca*^2 +^ -current	14.510^−9 ^mS.cm^−2^
*E*_*Ca*_	Equilibrium potential of *Ca*^2 +^ -current	140 mV
*τ*_*h*_	Parameter for *h*	10 ms
*θ*_*h*_	Parameter for *h*	−41 mV
*σ*_*h*_	Parameter for *h*	−0.5
*τ*_*m*_	Parameter for *m*	1.3 ms
*θ*_*m*_	Parameter for *m*	37 mV
*σ*_*m*_	Parameter for *m*	1
*V*_0_	Initial value for *V*	−65.0974
*n*_0_	Initial value for *n*	0.3162
*m*_0_	Initial value for *m*	4.5649.10^−45^
*h*_0_	Initial value for *h*	1
